# Axial length growth and the risk of developing myopia in European children

**DOI:** 10.1111/aos.13603

**Published:** 2017-12-19

**Authors:** Jan Willem Lodewijk Tideman, Jan Roelof Polling, Johannes R. Vingerling, Vincent W. V. Jaddoe, Cathy Williams, Jeremy A. Guggenheim, Caroline C. W. Klaver

**Affiliations:** ^1^ Department Ophthalmology Erasmus Medical Centre Rotterdam The Netherlands; ^2^ Department Epidemiology Erasmus Medical Centre Rotterdam The Netherlands; ^3^ Department Orthoptics University of Applied Science Utrecht The Netherlands; ^4^ School of Social and Community Medicine University of Bristol Bristol UK; ^5^ School of Optometry and Vision Sciences Cardiff University Cardiff UK

**Keywords:** axial length, children, growth curve, myopia

## Abstract

**Purpose:**

To generate percentile curves of axial length (AL) for European children, which can be used to estimate the risk of myopia in adulthood.

**Methods:**

A total of 12 386 participants from the population‐based studies Generation R (Dutch children measured at both 6 and 9 years of age; *N* = 6934), the Avon Longitudinal Study of Parents and Children (ALSPAC) (British children 15 years of age; *N* = 2495) and the Rotterdam Study III (RS‐III) (Dutch adults 57 years of age; *N* = 2957) contributed to this study. Axial length (AL) and corneal curvature data were available for all participants; objective cycloplegic refractive error was available only for the Dutch participants. We calculated a percentile score for each Dutch child at 6 and 9 years of age.

**Results:**

Mean (SD) AL was 22.36 (0.75) mm at 6 years, 23.10 (0.84) mm at 9 years, 23.41 (0.86) mm at 15 years and 23.67 (1.26) at adulthood. Axial length (AL) differences after the age of 15 occurred only in the upper 50%, with the highest difference within the 95th percentile and above. A total of 354 children showed accelerated axial growth and increased by more than 10 percentiles from age 6 to 9 years; 162 of these children (45.8%) were myopic at 9 years of age, compared to 4.8% (85/1781) for the children whose AL did not increase by more than 10 percentiles.

**Conclusion:**

This study provides normative values for AL that can be used to monitor eye growth in European children. These results can help clinicians detect excessive eye growth at an early age, thereby facilitating decision‐making with respect to interventions for preventing and/or controlling myopia.

## Introduction

Refractive errors such as myopia, hyperopia and astigmatism are the most common ocular disorders worldwide. The prevalence of these conditions varies with both age and geographic location (Laatikainen & Erkkilä 1980; Mantyjarvi 1983; Morgan et al. 2010; Tideman et al. 2016a). Myopia is most prevalent in Eastern Asia (Pan et al. 2015) and in the Western world (Vitale et al. 2009; Williams et al. 2015), whereas hyperopia is more prevalent in developing countries (Morgan et al. 2010).

Refractive error is the result of a mismatch between the various optical components of the eye, the most important of which are the cornea, the crystalline lens and the eye's AL. In the first few years of age, the cornea's refractive power is reduced; the lens also loses refractive power during childhood (Mutti et al. 1998; Iribarren et al. 2012). In contrast, AL increases during childhood and in the teenage years, leading to myopia if this growth in AL exceeds the eye's focal point (Zadnik et al. 2003). High myopia, which is defined as spherical equivalent (SE) of −6 D or worse, generally corresponds to AL ≥26 mm, which drastically increases the risk of severe complications later in life, including myopic maculopathy, retinal detachment and glaucoma (Curtin & Karlin 1971; Saw 2006; Tideman et al. 2016b). High myopia in adulthood usually has a myopia onset before the age of 10, which progresses during teenage years and early twenties (Möttönen et al. 1995; Fledelius 2000; Pärssinen et al. 2014; Tideman et al. 2017); therefore, the ability to identify young at‐risk children would provide clinicians the opportunity to apply preventative measures to minimize further increases in AL (Nordhausen et al. 2015). These measures can include changes in lifestyle (e.g. increasing outdoor exposure (He et al. 2015)), pharmacological agents such as atropine (Chia et al. 2016; Polling et al. 2016) and optical applications such as multifocal contact lenses (Turnbull et al. 2016).

Normative values as a function of age are available for a variety of measurements such as height, weight and birthweight, and these values are generally visualized using percentile curves. These curves are a powerful tool used by clinicians for sensitively detecting aberrant growth at an early age. Percentile curves for most body measurements, such as height and weight for gestational age, and height in childhood, have been generated using cross‐sectional data from extremely large cohorts (Niklasson et al. 1991; Schonbeck et al. 2013); however, no such normative data currently exist for ocular biometry components or refractive error.

The aim of this study was to generate a growth chart for AL based on large epidemiological cohorts of European children and adults. We assessed the risk of developing myopia and/or high myopia per percentile, and we examined how growth curves from Western Europe relate AL measurements in other geographic regions.

## Patients and Methods

### Study population

The study included three population‐based studies: the Generation R study, the ALSPAC and the RS‐III.

### The Generation R study

The Generation R study is a population‐based prospective cohort study of pregnant women and their subsequent children, conducted in Rotterdam, the Netherlands. The complete methodology for this study has been described elsewhere (Jaddoe et al. 2012; Kruithof et al. 2014). In brief, a total of 9778 pregnant women were included in the study, and their children were born from April 2002 through January 2006. At 6 and 9 years of age, the children were invited for an examination by trained nurses at a research centre. From the initial cohort, 6690 (68.4%) children participated in the physical examination at 6 years of age, and 5862 (60.0%) participated at 9 years of age. Follow‐up data regarding AL were available for 4787 children at both ages.

### The Avon longitudinal study of parents and children

Avon Longitudinal Study of Parents and Children (ALSPAC) is a prospective population‐based birth cohort study based in the former Avon health authority area in SouthWest England. This study was designed to investigate the determinants for development, health and disease in childhood and adulthood. Subject recruitment for this study has been described previously (Boyd et al. 2013). In brief, pregnant women with an expected date of delivery from April 1, 1991, through December 31, 1992, were eligible to participate, and 14 541 eligible women were recruited. These pregnancies resulted in 14 062 live births, and 13 988 of the infants were still alive at 1 year of age. Eye examinations were performed in these children from 7 years of age onwards, and ocular biometry measurements were included at age 15.

### The Rotterdam Study III

Rotterdam Study III (RS‐III) is a prospective, population‐based cohort study of subjects ≥45 years of age living in Ommoord, a suburb of Rotterdam, the Netherlands. In this study, researchers examined cardiovascular, endocrine, neurological, respiratory and ophthalmic outcomes. Baseline examinations – including best‐corrected visual acuity and refractive error measurements – were performed from 2006 through 2008. Axial length (AL) was measured in a random subset of the RS‐III cohort at baseline and in a different random subset during follow‐up examinations in 2011–2012 (Hofman et al. 2013).

### Ethical approval

Written informed consent was obtained from all participants or parents in all three cohorts.

The study protocols for the Generation R study and RS‐III were approved by the Medical Ethics Committee of the Erasmus Medical Centre, Rotterdam, the Netherlands. Ethics approval for the ALSPAC study was obtained from the Law and Ethics Committee and the respective local research ethics committees (http://www.bris.ac.uk/alspac/researchers/data-access/data-dictionary). All research was conducted in accordance with the Declaration of Helsinki.

### Data collection

In the Generation R and ALSPAC studies, ocular biometry was measured using a Zeiss IOLMaster 500 (Carl Zeiss, Jena, Germany or Welwyn Garden City, UK). In RS‐III, AL was measured using an A‐scan ultrasound device (Pacscan 300AP; Sonomed Escalon, MEyeTech GmbH, Hardegsen Germany) or LenStar device (Laméris Ootech, Haag‐Streit, UK). Corneal curvature was measured using a Topcon RM‐A2000 autorefractor (Topcon Optical Company, Tokyo, Japan). For measuring AL, five measurements were obtained per eye and were then averaged to obtain a mean AL value. For the corneal radius, three measurements of K1 and K2 were obtained per eye and averaged to obtain a mean corneal radius of curvature (CR). Axial length (AL)/CR ratio was calculated by dividing AL (in mm) by CR (in mm).

To calculate axial elongation and the change in corneal radius in mm/year, and the change in AL/CR ratio in mm/mm/year, the measurement at 6 years of age was subtracted from the measurement at 9 years of age, and divided by the number of years between the two measurements. Refractive error was available in Generation R at 9 years and in the RS‐III. In the Generation R cohort, automated cycloplegic refraction was measured in a random subsample at 9 years of age using a Retinomax‐3 device (Bon, Lübeck, Germany). At least 30 minutes prior to measuring refractive error, two drops (three with dark irises) of cyclopentolate (1%) were administered, and a pupil diameter ≥6 mm was required before SE was determined. Spherical equivalent (SE) was calculated as the average sphere + 1/2 cylinder for both eyes. In the RS‐III cohort, refraction was measured objectively using a Topcon RM‐A2000 (Topcon Optical company), and then subjectively adjusted with +0.25 D or −0.25 D steps, spherically as well as cylindrically to achieve the best possible visual acuity (VA). Myopia was defined as SE of ≤−0.5 D, emmetropia was defined as SE between −0.5 and +2.0 D, and hyperopia was defined SE ≥ +2.0 D. At the age of 6 years in Generation R, cycloplegic refractive error was only obtained when VA was worse than 0.2 LogMAR, detecting myopia ≤−0.5 but not hyperopia; we therefore did not use refractive error data at age 6 for analyses. In contrast, cycloplegic refractive error was collected in all 9‐year‐old, and non‐cycloplegic refraction was collected in all adults.

### Statistical methods

Average values of AL, CR and AL/CR were calculated. Differences between genders were analysed using the Student's *t*‐test or the chi‐square test. The association between biometry variables and SE were determined using linear regression models. For the growth curves of AL and AL/CR, we used the 2nd, 5th, 10th, 25th, 50th, 75th, 90th, 95th and 98th percentile values for the children in the Generation R and ALSPAC studies, with the measurements in the RS‐III cohort as the final refractive state in adults. Axial length (AL) was plotted against age, and an interpolation line was created between the matching percentiles of each age. Individual percentiles for AL at 6 and 9 years of age were calculated relative to the entire cohort, and the absolute difference between 6 and 9 years was calculated. To test for concordance of our results with other studies conducted in other geographic regions, we extracted data from 13 other population‐based and school‐based studies that were conducted in North America (Zadnik et al. 2003), Europe (Larsen 1971; Rudnicka et al. 2010; Li et al. 2015b), Asia (Li et al. 2011, 2015a; Iribarren et al. 2012; Hashemi et al. 2015; Jin et al. 2016; Lu et al. 2016) and Australia and Vanuatu (Garner et al. 1988; Ojaimi et al. 2005; Ip et al. 2008) for which gender‐stratified data were available. The association between SE and either AL or AL/CR ratio was determined using linear regression models and ordinary least squares linear regression models, with restricted cubic splines with three knots (the 10th, 50th and 90th percentiles) in the 9‐year‐old children in the Generation R cohort. All models were adjusted for both age and gender. Ordinary least squares linear regression models were generated using the program R; all other statistical analyses were performed using spss version 21.0 (IBM Corp., Armonk, NY, USA).

## Results

### Ocular biometry and refractive error

Analyses were performed at the cohort level. In the Generation R cohort, complete ocular biometry data were available for 6084 and 5295 children at 6 and 9 years of age, respectively. In the ALSPAC cohort, complete ocular biometry data were available for 2495 children 15 years of age. In the RS‐III cohort, data were available for 2957 adults with a mean age of approximately 57 years. The general demographic characteristics of all participants in all four age categories are shown in Table 1. In the children 6 and 9 years of age, mean (SD) AL was 22.36 (0.75) and 23.10 (0.84) mm, respectively. Axial length (AL) was 23.41 (0.86) mm in the 15‐year‐old and 23.67 (1.26) mm in the adults. Among all four cohorts, the minimum and maximum AL values were 17.54 and 30.12 mm, respectively. Mean (SD) CR was 7.77 (0.26) and 7.78 (0.26) mm in the 6‐year‐old and 9‐year‐old children, respectively, 7.82 (0.27) mm in the 15‐year‐old, and 7.74 (0.26) mm in the adults. Among all four cohorts, the minimum and maximum CR values were 6.91 and 9.61 mm, respectively. The mean (SD) AL/CR ratio was 2.88 (0.08) in the 6‐year‐old and 3.05 (0.15) in the adults; among all four cohorts, the minimum and maximum AL/CR values were 2.38 and 4.07, respectively. On average, the females in each age group had significantly shorter AL, steeper CR and lower AL/CR ratios compared to the males in their respective age groups (p < 0.001). The gender‐stratified mean and SD values for general and ocular characteristics are shown in Table 1. Height had the strongest correlation with AL in the 6‐year‐old group (*β *= 0.028; p < 0.001), and this correlation decreased slightly – but remained significant – in the 9‐year‐old group (*β *= 0.024; p < 0.001). No significant difference in height was found between the refractive error groups in boys [one‐way analysis of variance (anova) p = 0.40] as well as girls (anova p = 0.24).

**Table 1:** General and ocular characteristics of the four study cohorts

	All	Male	Female	p‐value^b^
Generation R at 6 years of age (*N* = 6084)				
Age in years	6.17 (0.52)	6.18 (0.55)	6.16 (0.50)	0.03
Gender, *N* (%)	6084 (100)	3033 (49.9)	3051 (50.1)	NA
European ethnicity, *N* (%)	4089 (67.2)	2023 (66.7)	2066 (67.7)	0.41
Height in cm	119 (6)	120 (6)	119 (6)	<0.001
AL in mm	22.36 (0.75)	22.63 (0.73)	22.09 (0.7)	<0.001
Corneal radius in mm	7.77 (0.26)	7.84 (0.26)	7.70 (0.24)	<0.001
AL/CR ratio	2.88 (0.08)	2.89 (0.08)	2.87 (0.08)	<0.001
Generation R at 9 years of age (*N* = 5296)				
Age in years	9.79 (0.33)	9.80 (0.36)	9.77 (0.31)	0.02
Gender, *N* (%)	5296 (100)	2617 (49.4)	2679 (50.6)	NA
European ethnicity, *N* (%)	3770 (71.2)	1842 (70.4)	1928 (72.0)	0.21
Height in cm	142 (6)	142 (6)	141 (7)	0.05
AL in mm	23.10 (0.84)	23.36 (0.82)	22.84 (0.78)	<0.001
Corneal radius in mm	7.78 (0.26)	7.85 (0.26)	7.72 (0.24)	<0.001
AL/CR ratio	2.97 (0.09)	2.98 (0.10)	2.96 (0.09)	<0.001
SE in dioptres^a^	0.74 (1.30)	0.73 (1.28)	0.75 (1.31)	0.66
ALSPAC cohort (*N* = 2495)				
Age in years	15.47 (0.32)	15.45 (0.29)	15.49 (0.34)	0.001
Gender, *N* (%)	2495 (100)	1167 (46.7)	1328 (53.3)	NA
European ethnicity, *N* (%)	2447 (98.1)	1145 (98.1)	1302 (98.0)	0.79
Height in cm	169 (8)	175 (7)	165 (6)	<0.001
AL in mm	23.41 (0.86)	23.68 (0.88)	23.18 (0.84)	<0.001
Corneal radius in mm	7.82 (0.27)	7.88 (0.27)	7.77 (0.25)	<0.001
AL/CR ratio	2.99 (0.1)	3.01 (0.1)	2.98 (0.10)	<0.001
RS‐III cohort (*N* = 2957)				
Age in years	56.8 (6.4)	56.8 (6.3)	56.8 (6.3)	0.83
Gender, *N* (%)	2957 (100)	1290 (43.6)	1667 (56.4)	NA
European ethnicity, *N* (%)	2745 (92.8)	1215 (94.2)	1530 (91.8)	0.01
Height in cm	170.5 (10)	178 (6)	164 (7)	<0.001
AL in mm	23.67 (1.26)	23.99 (1.26)	23.42 (1.20)	<0.001
Corneal radius in mm	7.74 (0.26)	7.81 (0.25)	7.69 (0.25)	<0.001
AL/CR ratio	3.05 (0.15)	3.07 (0.16)	3.04 (0.15)	<0.001
SE in dioptres	−0.31 (2.5)	−0.39 (2.5)	−0.26 (2.5)	0.16

Except where indicated otherwise, all data are presented as the mean (SD).

AL = axial length, CR = corneal radius of curvature, SE = spherical equivalent, RS‐III = Rotterdam Study III.

a p‐values were calculated using the Student's *t*‐test or the chi‐square test.

b *N* = 2408 (1204 males and 1204 females).

Refractive error had a relatively narrow distribution in both the 9‐year‐old and the adults (Fig. S1), with mean SE values of +0.74 D (SD: 1.30; range: −9.8 D to +8.3 D) and −0.31 D (SD: 2.53; range: −13.8 D to +9.1 D), respectively. At 9 years of age, there was no significant difference in SE between boys and girls (mean SE was +0.73 D and +0.75 D, respectively; p = 0.66); we also found no significant difference between the adult males and females (−0.39 D versus −0.26 D, respectively; p = 0.16). Among the 9‐year‐old children, 11.4% (*N* = 274) and 8.4% (*N* = 203) had myopia and hyperopia, respectively; among the adults, 37.0% (*N* = 1093) and 11.9% (*N* = 352) had myopia and hyperopia, respectively.

**Figure 1:**
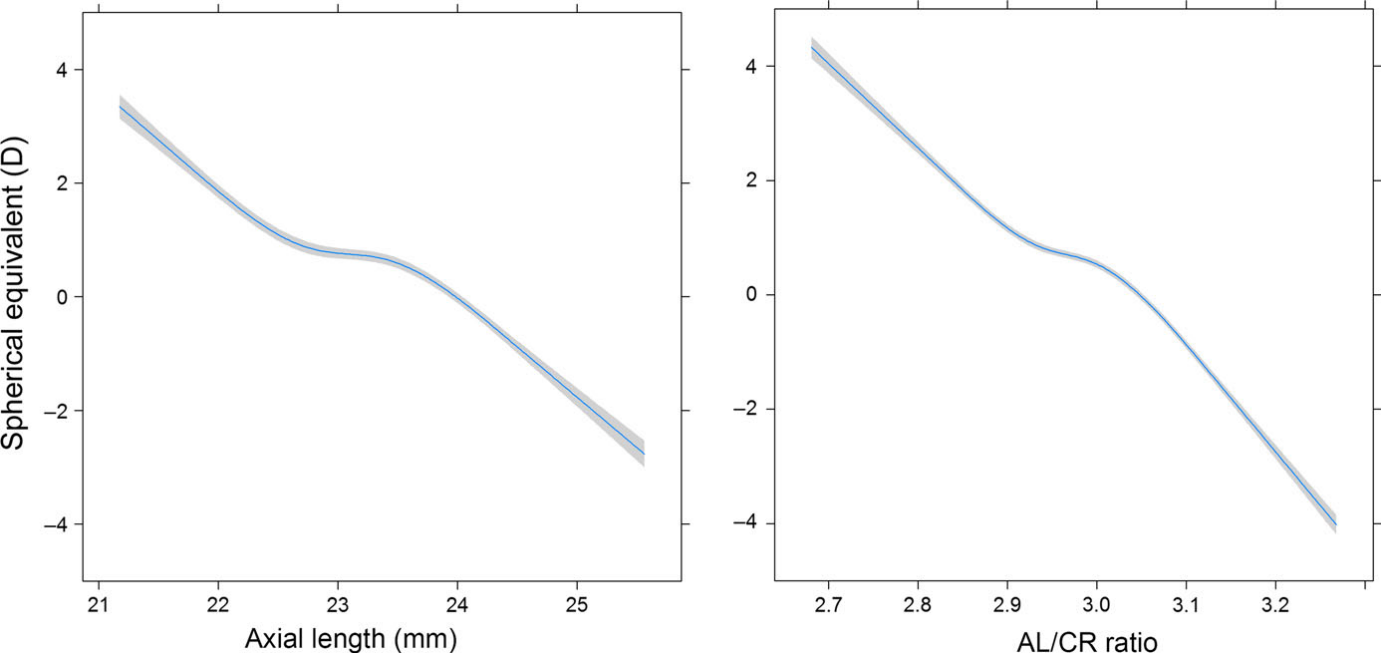
Association between spherical equivalent (in dioptres) and axial length (AL) (in mm; left) and AL/corneal radius of curvature ratio (right) at 9 years of age. The mean and 95% CI were adjusted for age, gender and height.

Table 2 summarizes the differences in ocular biometry and the association between SE and the various refractive error groups in the Generation R and RS‐III cohorts. Our analysis revealed that SE was inversely correlated with both AL and the AL/CR ratio in both the Generation R (Fig. 1) and RS‐III cohorts. Interestingly, the relationship between SE and AL/CR ratio was non‐linear (quadratic term p < 0.001). The correlation between SE and both AL and AL/CR ratio was weakest in the emmetropic participants and strongest in the myopic participants (Table 2).

**Table 2:** Ocular biometry and correlation with SE in children and adults

	Children at 9 years of age (*N* = 2408)		Adults ≥45 years of age (*N* = 2957)	
	Mean (SD; 90% range)	*β* (95% CI) of association with SE	Mean (SD, 90% range)	*β* (95% CI) of association with SE
AL (mm)				
All	23.10 (0.81; 21.79 to 24.42)	−1.06 (−1.12 to −1.01)	23.67 (1.26; 21.82 to 25.90)	−1.61 (−1.66 to −1.56)
Hyperopia	22.08 (0.69; 21.20 to 23.28)	−0.82 (−1.02 to −0.62)	22.30 (0.90; 20.70 to 23.72)	−1.04 (−1.16 to −0.91)
Emmetropia	23.08 (0.67; 22.02 to 24.23)	−0.25 (−0.28 to −0.21)	23.30 (0.85; 21.95 to 24.71)	−0.23 (−0.23 to −0.19)
Myopia	23.98 (0.83; 22.75 to 25.37)	−0.98 (−1.15 to −0.82)	24.62 (1.19; 22.86 to 26.58)	−1.24 (−1.34 to −1.16)
p‐value	<0.001		<0.001	
CR (mm)				
All	7.78 (0.25; 7.38 to 8.22)	0.70 (0.49 to 0.91)	7.74 (0.26; 7.33 to 8.18)	1.10 (0.74 to 1.46)
Hyperopia	7.80 (0.26; 7.38 to 8.26)	1.11 (0.52 to 1.69)	7.79 (0.25; 7.39 to 8.23)	0.13 (−0.47 to 0.74)
Emmetropia	7.79 (0.25; 7.39 to 8.22)	0.19 (0.01 to 0.29)	7.75 (0.26; 7.33 to 8.20)	0.12 (−0.13 to 0.24)
Myopia	7.73 (0.25; 7.38 to 8.26)	0.63 (−0.05 to 1.31)	7.72 (0.26; 7.30 to 8.15)	0.44 (−0.05 to 0.93)
p‐value	<0.001		0.008	
AL/CR ratio				
All	2.97 (0.09; 2.84 to 3.13)	−11.56 (−11.89 to −11.23)	3.05 (1.51; 2.83 to 3.32)	−14.43 (−14.73 to −14.13)
Hyperopia	2.83 (0.08; 2.40 to 3.01)	−9.77 (−10.91 to −8.62)	2.86 (0.11; 2.69 to 3.02)	−9.94 (−10.96 to −8.92)
Emmetropia	2.96 (0.06; 2.87 to 3.06)	−4.43 (−4.76 to −4.11)	3.01 (0.08; 2.87 to 3.14)	−3.35 (−3.73 to −2.97)
Myopia	3.10 (0.09; 2.97 to 3.25)	−11.07 (−12.24 to −9.90)	3.19 (0.14; 3.00 to 3.42)	−12.43 (−13.03 to −11.84)
p‐value	<0.001		<0.001	
AL growth (mm/year)				
All	0.21 (0.08; 0.11 to 0.37)	−10.54 (−11.05 to −10.04)	NA	NA
Hyperopia	0.15 (0.06; 0.06 to 0.26)	−5.01 (−7.31 to −2.71)	NA	NA
Emmetropia	0.19 (0.05; 0.12 to 0.29)	−3.64 (−4.07 to −3.21)	NA	NA
Myopia	0.34 (0.11; 0.17 to 0.53)	−5.86 (−7.30 to −4.44)	NA	NA
p‐value	<0.001		NA	
CR growth (mm/year)				
All	0.004 (0.01; −0.010 to 0.015)	1.46 (−3.60 to 6.52)	NA	NA
Hyperopia	0.003 (0.01; −0.010 to 0.015)	4.80 (−7.79 to 17.40)	NA	NA
Emmetropia	0.004 (0.01; −0.009 to 0.015)	−0.42 (−2.69 to 1.85)	NA	NA
Myopia	0.003 (0.01; −0.013 to 0.015)	−3.34 (−21.07 to 14.39)	NA	NA
p‐value	0.37		NA	
AL/CR change (units per year)				
All	0.025 (0.011; 0.012 to 0.046)	−72.73 (−76.55 to −68.92)	NA	NA
Hyperopia	0.018 (0.010; 0.005 to 0.034)	−31.97 (−47.33 to −16.60)	NA	NA
Emmetropia	0.023 (0.008; 0.013 to 0.037)	−22.82 (−25.84 to −19.80)	NA	NA
Myopia	0.043 (0.014; 0.021 to 0.068)	−41.31 (−51.99 to −30.63)	NA	NA
p‐value	<0.001		NA	

Except where indicated otherwise, all data are presented as the mean (SD). Sample size in the refractive error categories at 9‐year‐old: hyperopia, *N* = 203; emmetropia, *N* = 1926; myopia, *N* = 279. Sample size in the refractive error categories in the adults: hyperopia, *N* = 352; emmetropia, *N* = 1512; myopia *N* = 1093. In the regression models, SE was used as the dependent variable, and the ocular biometry measurements were used as the independent variable. The models were adjusted for age, gender, ethnicity and height. p‐values reflect the differences in ocular biometry measurements between the refractive groups and were calculated using an anova.

AL = axial length, CR = corneal radius of curvature, NA = not applicable (no follow‐up data were available), SE = spherical equivalent.

In addition, SE was significantly correlated with CR. On average, the myopic children had a steeper CR (7.73 mm) compared to both the emmetropic (7.79 mm; p < 0.001) and hyperopic (7.80 mm; p < 0.001) children. Similar results were obtained in the adult cohort (Table 2).

Longitudinal changes in AL were also measured in the Generation R cohort between the 6‐year‐old and 9‐year‐old children. On average, AL increased by 0.21 mm/year (SD: 0.08 mm/year), and the AL/CR ratio increased by 0.025 units/year (SD: 0.011 units per year). The myopic children had more rapid eye growth rate (0.34 mm/year) than both the emmetropic (0.19 mm/year; p < 0.001) and hyperopic (0.15 mm/year; p < 0.001) children. At 9 years of age, the increases in AL and AL/CR ratio were significantly associated with a shift in refractive error towards increased myopia; this result was present in all refractive error categories. We found no significant change in CR from 6 to 9 years of age (Table 2).

### AL growth curves

**Figure 2:**
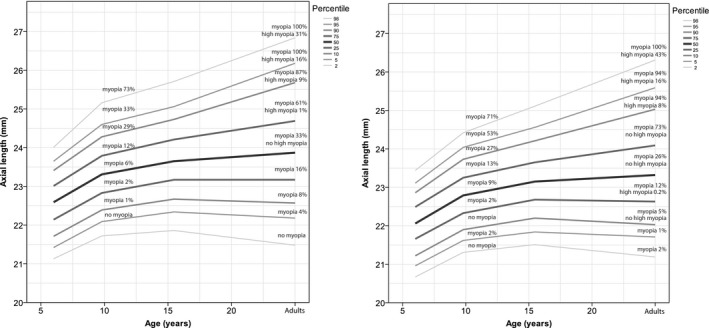
Growth chart depicting axial length (in mm) versus age for European study subjects, males (left) and females (right), with the risk of myopia in adulthood. The myopia percentage represents the proportion of myopia in halfway above and below the percentage line.

Figure 2 shows the growth chart for AL versus age in percentiles. From 6 to 9 years of age, all of the percentiles examined increased in AL; however, none of the percentiles below the median increased further after the age of 15. In particular, the lowest percentiles of AL increased relatively little after the age of 6, and the 5th percentile values changed by <1 mm with age. The AL of all of the median and above‐median percentiles increased until adulthood. The median percentile in the male participants increased by 1.28 mm (22.59 mm versus 23.87 mm at 6 years of age and adulthood, respectively); and the 95th percentile increased by 2.5 mm [23.65 mm versus 26.18 mm at 6 years of age and adulthood, respectively, (Fig. 2 and Table S1a)]. Similar results were observed for AL in the female participants (Fig. 2 and Table S1b) and for the AL/CR ratio in both genders (Fig. S2). The above‐median percentiles of AL were associated with a >50% risk of developing myopia in adulthood age; moreover, the highest 10th percentile was associated with a 97% risk of myopia and a 23% risk of high myopia. Corneal radius of curvature (CR) was relatively consistent across all age groups (Fig. S3).

**Figure 3:**
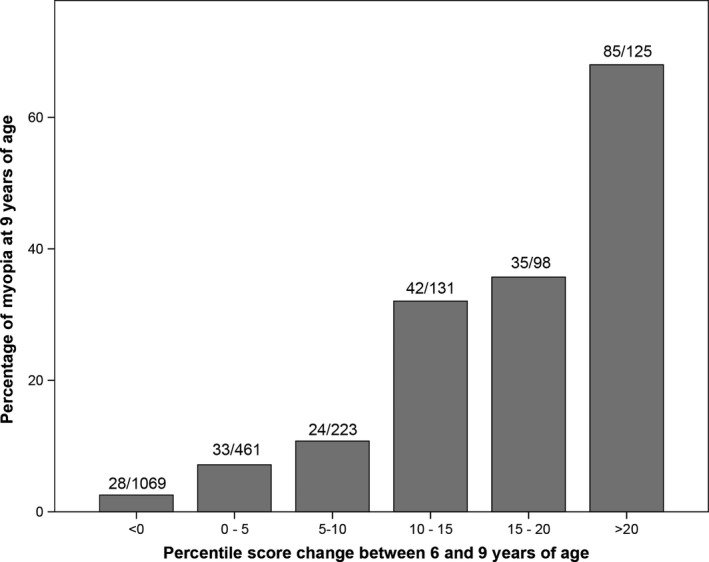
The change in percentile score of axial length between 6 and 9 years of age (*x*‐axis) and the percentage of myopia at 9 years of age (*y*‐axis).

The median absolute difference in AL was 5.6 percentiles (IQR: 2.4–11.2), indicating that a given child's percentile at age 6 is a reliable predictor of that child's percentile at age 9. Moreover, we found a significant correlation in percentile position between 6 and 9 years of age (Spearman correlation coefficient: 0.92; p < 0.001). Higher change in percentile position was highly correlated to myopia prevalence (Fig. 3). Of the 354 children who had an increase in percentile score of ≥10, 45.8% (*N* = 162) were myopic at 9 years of age; in contrast, only 4.8% (85/1781) of the children who had an increase in percentile score <10 were myopic at 9 years of age.

### Support for our growth curves based on previous publications

**Figure 4:**
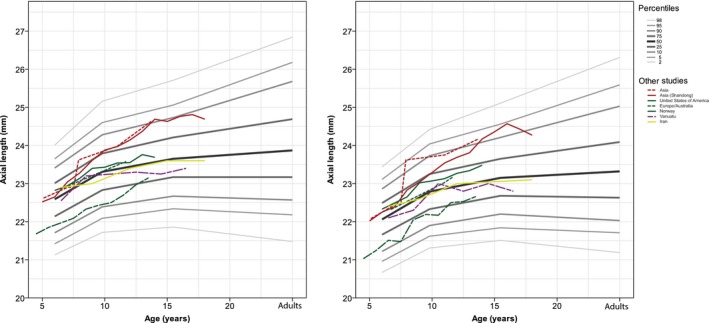
Axial length is plotted against age for male (left) and female (right) children from various geographic locations. For comparison, the data from the present study are copied from Fig. 2 and are shown here in grey. Gender‐stratified data were collected from Australia, Europe, the United States, Iran, Vanuatu and Norway. The European and Australian children were clustered as being predominantly of European descent. Solid lines are single studies, dashed line multiple studies from the same geographic regions and irregular dashed lines single studies published before 1990.

Finally, we used gender‐stratified AL measurements obtained from published population‐based and school‐based studies to confirm our growth curves. As shown in Fig. 4, the median AL growth rates in studies of European children were similar to our own median values. The mean AL value in Asian populations was larger after 7 years of age. In addition, the mean AL values in the children measured in both Vanuatu study and in an older study of Norwegian children were smaller than our median value (Larsen 1971; Garner et al. 1988).

## Discussion

The aim of this study was to provide normative growth values for ocular biometry and the associated risk of developing myopia in European children. Our analysis revealed that median AL increased with age until 15 years of age, after which AL continued to increase into adulthood in the top 50th percentile. Corneal radius of curvature (CR) was relatively similar across age groups, with only a slightly smaller corneal radius in the adult cohort. At 9 years of age, the children in the European cohorts were generally emmetropic, with an average SE of +0.74 D, and 11.4% of these children were already myopic. The correlation between SE and AL/CR ratio and was not linear as a whole; rather, it was weaker around the emmetropic values. This was likely due to compensation by other optical features such as the crystalline lens and anterior chamber depth (Iribarren 2015).

Our study has several strengths. First, we included more than 12 000 measurements of ocular biometry in European children and adults in four discrete age categories. Second, the studies from which we collected our data used autorefraction to measure refractive error. Third, the age ranges of the children were extremely narrow, allowing for highly robust analysis. Finally, the data were stratified by gender.

Despite these strengths, several possible weaknesses warrant discussion. First, the ALSPAC study involving 15‐year‐old children was conducted in the UK, whereas the Generation R and RS‐III studies were conducted in the Netherlands; therefore, geographic and/or other factors may have affected our analysis. Second, we lacked a study population of young adults, and actual measurements of refractive error for ages 20–25 years would have corrected for small alternations of AL changes from early to late adulthood, whereas most of the axial elongation will occur between 15 and 25 years of age (Hashemi et al. 2016). Third, the birth years differed among the three cohorts, and younger cohorts may have a higher risk of myopia in adulthood compared to older cohorts (Vitale et al. 2009; Williams et al. 2015). Such a cohort effect may have led to an underestimation of the upward trend of the growth curve at age 15 and older. Fourth, differences in the instruments used (e.g. IOLMaster versus keratometry/A‐scan ultrasonography) for the various cohorts may have generated a systematic error in biometry measurements. Although AL measurements do not differ between instruments, CR values can differ by up to 0.03 mm between Topcon Keratometry and IOLMaster (Buckhurst et al. 2009; Jasvinder et al. 2011; Kolodziejczyk et al. 2011; Huang et al. 2012; Wang et al. 2012; Guler et al. 2016). Lastly, the published studies predominantly reported mean AL values, rather than median AL values. However, this likely had only had a slight effect on the trajectories, as the difference mean and median AL values were relatively low (0.03–0.12 mm) in all of our study cohorts.

Our findings are similar to other cohort data in several respects. First, we observed a gender difference in AL, CR and AL/CR ratio, which is consistent with previous observations (Ojaimi et al. 2005; Rudnicka et al. 2010; Li et al. 2015a,2015b; Pärssinen & Kauppinen 2016). In addition, we found that AL increased more rapidly in the myopic children than in the children with hyperopia, a finding consistent with the Northern Ireland Childhood Errors of Refraction (NICER) study (Breslin et al. 2013). We also compared the AL growth rates in our study with data obtained from other geographic regions and found several interesting ethnic and cohort effects. For example, children in East Asia generally have higher AL after the age of 6 years compared to both European and Iranian children, reflecting higher risk for developing myopia (Ojaimi et al. 2005; Rudnicka et al. 2010; Hashemi et al. 2015; Li et al. 2015b). Compared to the 6‐year‐old children in our Dutch study, 3‐year‐old Asian children have shorter AL and lower AL/CR ratios but similar CR values (Foo et al. 2016). At 5 years of age, children in Singapore had similar AL values as the 6‐year‐old children in our study (Li et al. 2011); however, at 8 years of age, the children in Singapore had longer AL values and higher AL/CR ratios than our 9‐year‐old children. In contrast, compared with our results, Northern European children in a study conducted in 1971 had lower AL values at all ages (Larsen 1971), which can be caused by a lower myopia prevalence as well as a lower body height, or a combination of these.

The prevalence of myopia among European children has only been examined in relatively few studies (Laatikainen & Erkkilä 1980; Mantyjarvi 1983; Pärssinen 2012). The multi‐ethnic Child Heart and Health Study in England (CHASE) study in the UK reported a prevalence of 11.9% (≤−0.50 D) at approximately 11 years of age (Rudnicka et al. 2010), and the NICER study in Northern Ireland reported a prevalence of 17.7% (≤−0.50 D) at approximately 13 years of age (O'Donoghue et al. 2015). The multi‐ethnic Collaborative Longitudinal Evaluation of Ethnicity and Refractive Error (CLEERE) study conducted in the US found a prevalence of 11.6% (≤−0.75 D in both meridians) in 10‐year‐old (Zadnik et al. 2003), and the Australian Sydney Myopia Study found a prevalence of 11.9% (≤−0.50 D) in 13‐year‐old (Ip et al. 2008). These values are similar to the prevalence of 11.4% that we found in our Dutch cohort of 9‐year‐old. We and others have found that height is associated with AL, and this needs to be taken into account when interpreting the growth curves.

Interestingly, our analysis revealed a large difference in eye growth between children at risk for developing myopia and children with low risk; specifically, the rate of eye growth was twice as high in the children who developed myopia compared to the children who remained hyperopic. Follow‐up studies are needed to determine whether children born after 2010 have a steeper growth curve than suggested by our growth chart. In addition, the growth curves can be improved further by focussing on children who differ in ages from those in our study, thereby providing complementary data.

## Conclusions

Our normative data regarding AL may serve as a key instrument for monitoring eye growth in children with progressive myopia in European and other populations. Paediatric ophthalmologists, optometrists and orthoptists can use these charts to determine whether a child's AL is above average for his/her age, and this information can be used to estimate the risk of developing high myopia. In addition, children with a rate of AL growth higher than expected based on their percentile line can be identified relatively early, allowing these children to benefit from the increasing number of therapeutic options for preventing myopia.

## Supporting information


**Figure S1.** Distribution of refractive error at age 9 years (left) and in adults (right).
**Figure S2.** AL/CR as a function of age in boys (left) and girls (right).
**Figure S3.** CR as a function of age boys (left) and girls (right).
**Table S1.** (a) Percentiles of axial length, corneal radius and AL/CR ratio in 6 and 9 year old European boys and (b) Percentiles of axial length, corneal radius and AL/CR ratio in 6 and 9 year old European boys.
