# Supplementary material for: Axial length growth and the risk of developing myopia in European children
Source: Acta Ophthalmol. 2017 Dec 19;96(3):301–9. doi: 10.1111/aos.13603 (PMC6002955; doi:10.1111/aos.13603)
Supplement: Supplementary file 1 — Figure S1. Distribution of refractive error at age 9 years (left) and in adults (right). Figure S2. AL/CR as a function of age in boys (left) and girls (right). Figure S3. CR as a function of age boys (left) and girls (right). Table S1. (a) Percentiles of axial length, corneal radius and AL/CR ratio in 6 and 9 year old European boys and (b) Percentiles of axial length, corneal radius and AL/CR ratio in 6 and 9 year old European boys. [file AOS-96-301-s001.docx]

**SUPPLEMENTS**

**Axial length growth and the risk of developing myopia in European children**

J. Willem L. Tideman^1,2^, MSc, MD, Jan Roelof Polling^1,3^, BSc Johannes R. Vingerling^1^, MD, PhD, Vincent W. V. Jaddoe^2^, MD, PhD, Cathy Williams^4^, MD, PhD, Jeremy A. Guggenheim^5^, PhD, Caroline C.W. Klaver^1,2^_,_ MD, PhD

^1^Department Ophthalmology, Erasmus Medical Centre, Rotterdam, the Netherlands; ^2^Department Epidemiology, Erasmus Medical Centre, Rotterdam, the Netherlands; ^3^Department Orthoptics, School of Applied Science, Utrecht, the Netherlands; ^4^School of Social and Community Medicine, University of Bristol, Bristol, UK; ^5^School of Optometry and Vision Sciences, Cardiff University, Cardiff, UK

**Correspondence**:

Prof. Caroline C.W. Klaver, MD, PhD; Erasmus Medical Center NA2808; PO Box 5201, 3008 AE Rotterdam, the Netherlands.

E-mail: c.c.w.klaver@erasmusmc.nl

**Supplementary Table S1a.** Percentiles of axial length, corneal radius and AL/CR ratio in 6 and 9 year old European boys

| **Percentile** | **AL** | **CR** | **AL/CR ratio** |
| --- | --- | --- | --- |
| **6 years visit (N = 1965)** | |  |  |
| 2 | 21.13 | 7.33 | 2.71 |
| 5 | 21.42 | 7.42 | 2.75 |
| 10 | 21.71 | 7.52 | 2.79 |
| 25 | 22.14 | 7.68 | 2.84 |
| 50 | 22.59 | 7.84 | 2.89 |
| 75 | 23.01 | 8.00 | 2.92 |
| 90 | 23.41 | 8.16 | 2.96 |
| 95 | 23.65 | 8.27 | 2.99 |
| 98 | 24.01 | 8.39 | 3.03 |
| **9 years visit (N = 1842)** |  |  |  |
| 2 | 21.72 | 7.34 | 2.77 |
| 5 | 22.09 | 7.43 | 2.84 |
| 10 | 22.39 | 7.53 | 2.87 |
| 25 | 22.83 | 7.69 | 2.92 |
| 50 | 23.31 | 7.84 | 2.97 |
| 75 | 23.79 | 8.02 | 3.02 |
| 90 | 24.28 | 8.17 | 3.07 |
| 95 | 24.60 | 8.27 | 3.12 |
| 98 | 25.16 | 8.41 | 3.20 |
| **15 years (ALSPAC; N = 1145)** | | | |
| 2 | 21.86 | 7.36 | 2.80 |
| 5 | 22.34 | 7.48 | 2.85 |
| 10 | 22.67 | 7.57 | 2.90 |
| 25 | 23.17 | 7.70 | 2.95 |
| 50 | 23.65 | 7.86 | 3.00 |
| 75 | 24.21 | 8.05 | 3.06 |
| 90 | 24.73 | 8.25 | 3.12 |
| 95 | 25.06 | 8.31 | 3.16 |
| 98 | 25.71 | 8.46 | 3.26 |
| **45+ years visit (RS III; N = 1215)** | | | |
| 2 | 21.48 | 7.29 | 2.76 |
| 5 | 22.18 | 7.40 | 2.83 |
| 10 | 22.57 | 7.50 | 2.90 |
| 25 | 23.17 | 7.64 | 2.97 |
| 50 | 23.87 | 7.81 | 3.05 |
| 75 | 24.69 | 7.97 | 3.16 |
| 90 | 25.68 | 8.14 | 3.28 |
| 95 | 26.18 | 8.26 | 3.35 |
| 98 | 26.84 | 8.35 | 3.44 |

**Supplementary Table S1b.** Percentiles of axial length, corneal radius and AL/CR ratio in 6 and 9 year old European girls

| **Percentile** | **AL** | **CR** | **AL/CR ratio** |
| --- | --- | --- | --- |
| **6 years visit (N = 2018)** | |  |  |
| 2 | 20.67 | 7.22 | 2.70 |
| 5 | 20.96 | 7.32 | 2.75 |
| 10 | 21.22 | 7.41 | 2.78 |
| 25 | 21.66 | 7.54 | 2.82 |
| 50 | 22.06 | 7.70 | 2.87 |
| 75 | 22.49 | 7.85 | 2.91 |
| 90 | 22.86 | 8.00 | 2.95 |
| 95 | 23.11 | 8.11 | 2.97 |
| 98 | 23.44 | 8.21 | 3.00 |
| **9 years visit (N = 1928)** | | | |
| 2 | 21.31 | 7.24 | 2.77 |
| 5 | 21.62 | 7.34 | 2.82 |
| 10 | 21.90 | 7.42 | 2.86 |
| 25 | 22.33 | 7.56 | 2.91 |
| 50 | 22.79 | 7.72 | 2.95 |
| 75 | 23.25 | 7.88 | 3.00 |
| 90 | 23.73 | 8.02 | 3.05 |
| 95 | 24.04 | 8.13 | 3.09 |
| 98 | 24.42 | 8.23 | 3.17 |
| **15 years visit (ALSPAC; N = 1302)** | | | |
| 2 | 21.51 | 7.27 | 2.77 |
| 5 | 21.84 | 7.37 | 2.84 |
| 10 | 22.20 | 7.46 | 2.87 |
| 25 | 22.68 | 7.61 | 2.93 |
| 50 | 23.15 | 7.76 | 2.98 |
| 75 | 23.65 | 7.93 | 3.03 |
| 90 | 24.21 | 8.10 | 3.10 |
| 95 | 24.56 | 8.21 | 3.14 |
| 98 | 25.11 | 8.31 | 3.23 |
| **RS III 45+ years visit (N = 1530)** | | | |
| 2 | 21.19 | 7.18 | 2.77 |
| 5 | 21.71 | 7.29 | 2.83 |
| 10 | 22.03 | 7.37 | 2.88 |
| 25 | 22.63 | 7.53 | 2.95 |
| 50 | 23.32 | 7.68 | 3.03 |
| 75 | 24.09 | 7.85 | 3.13 |
| 90 | 25.03 | 8.02 | 3.25 |
| 95 | 25.59 | 8.11 | 3.32 |
| 98 | 26.31 | 8.22 | 3.40 |


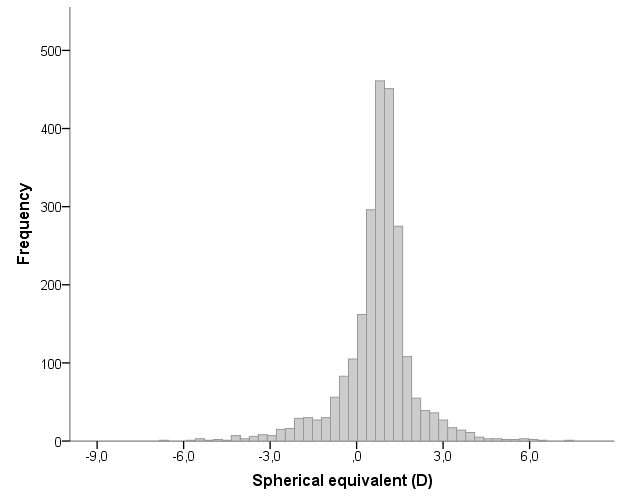

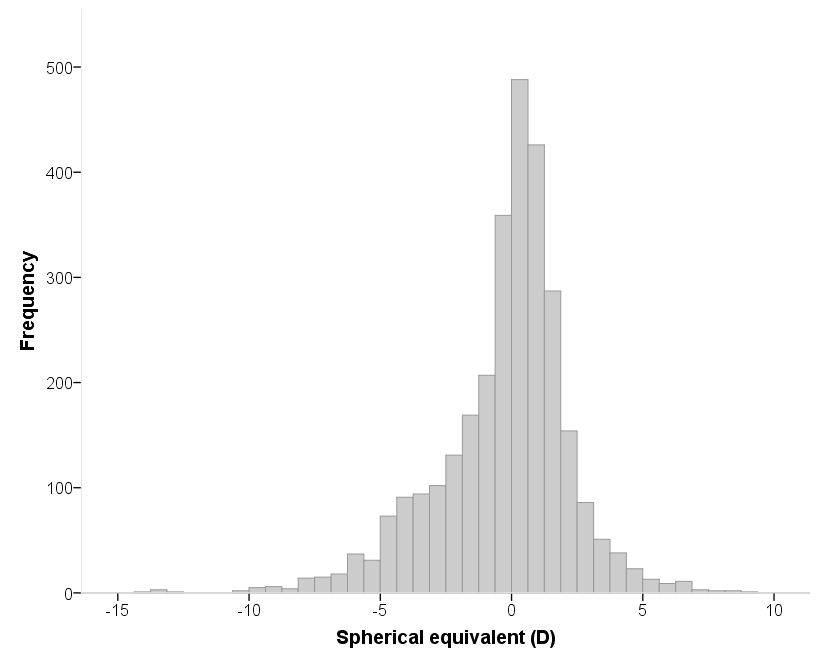
 **Supplementary Figure S1**. Distribution of refractive error at age 9 years (left) and in adults (right)

**Supplementary Figure S2.** AL/CR as a function of age in boys (left) and girls (right)


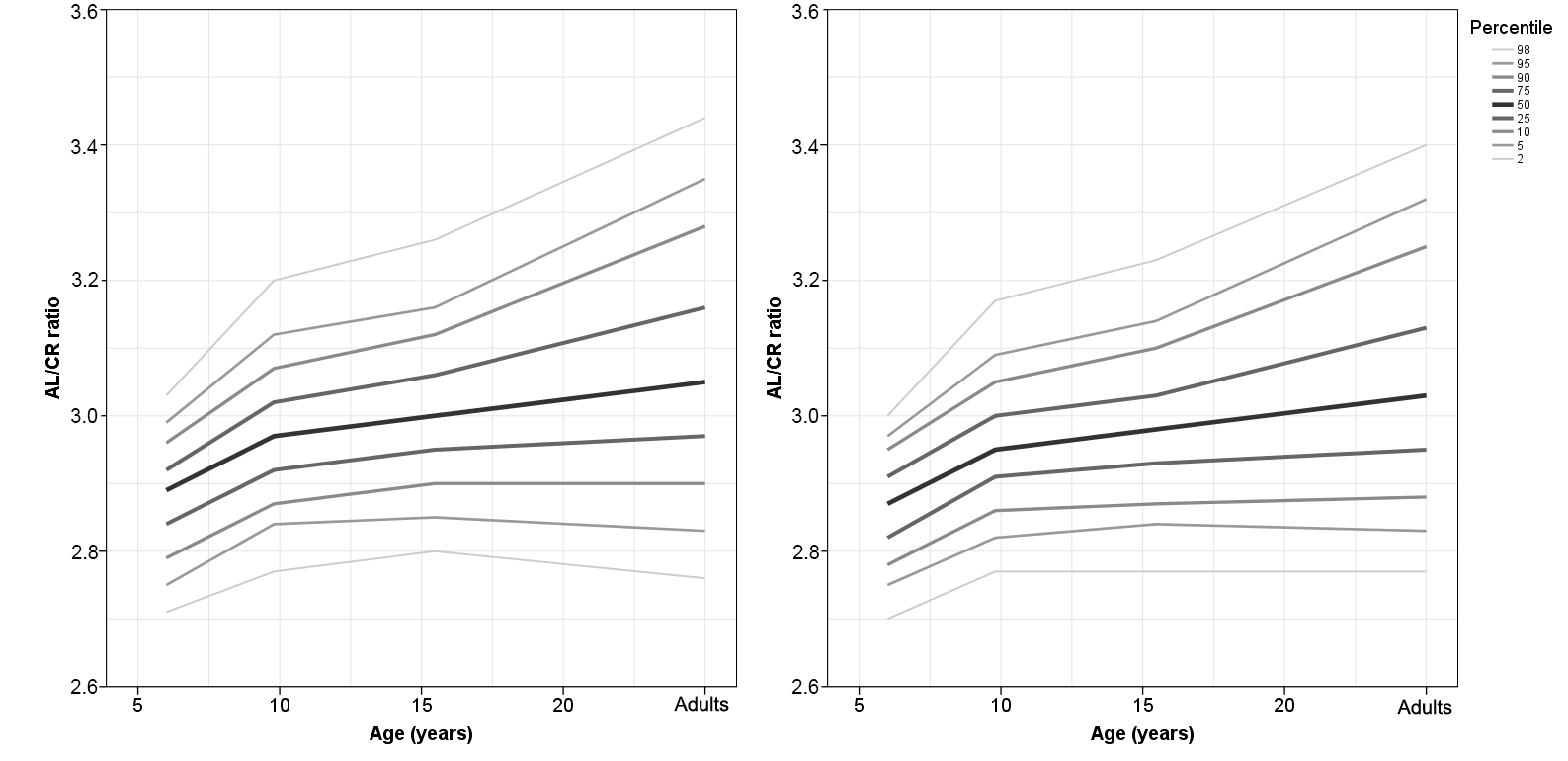


**Supplementary Figure S3.** CR as a function of age boys (left) and girls (right)

**
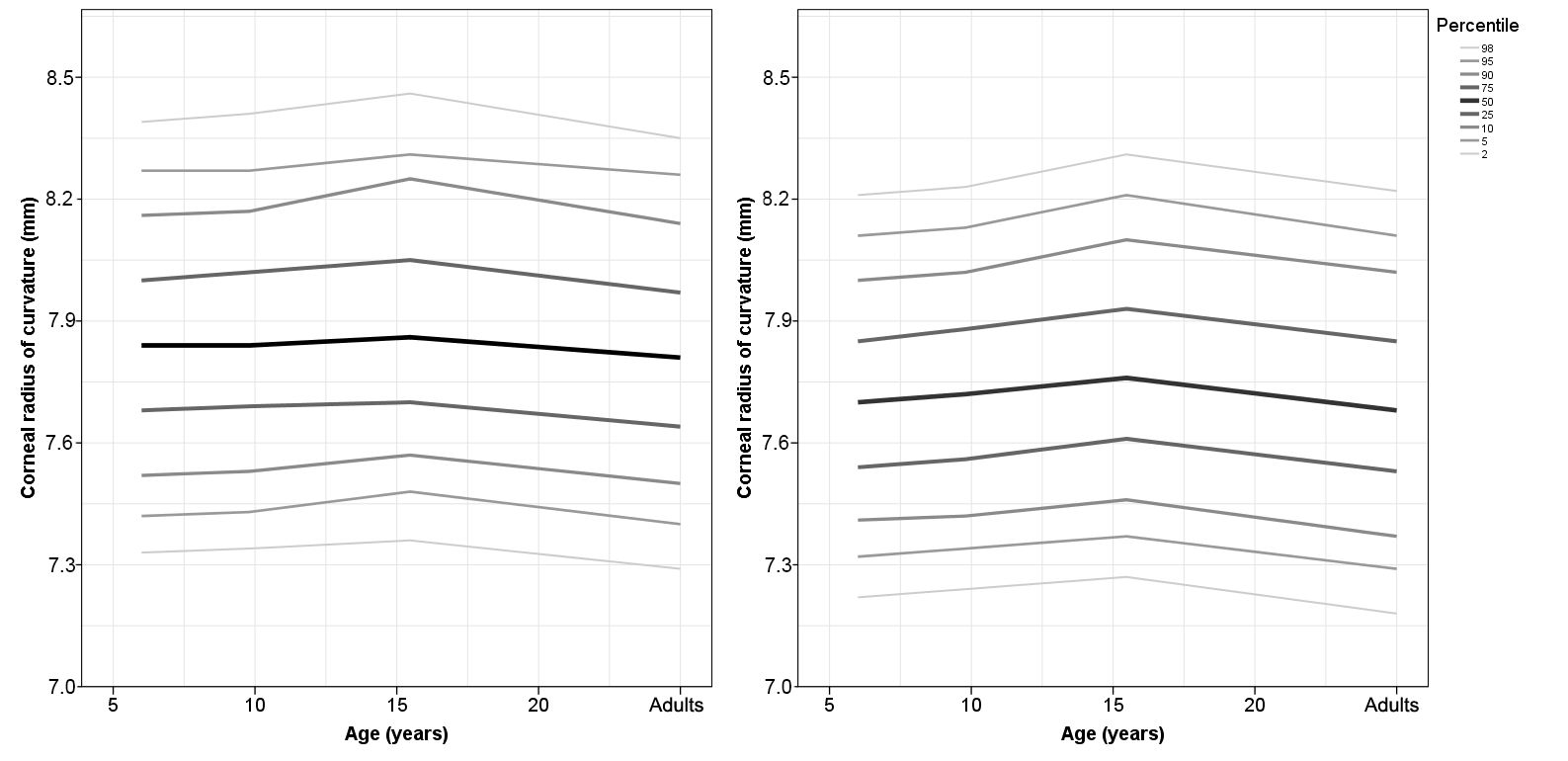
**
